# Cisplatin shows greater efficacy than gemcitabine when combined with nab-paclitaxel in metastatic triple-negative breast cancer

**DOI:** 10.1038/s41598-019-39314-y

**Published:** 2019-03-05

**Authors:** Yi Li, Yannan Zhao, Chengcheng Gong, Yizhao Xie, Xichun Hu, Jian Zhang, Leiping Wang, Sheng Zhang, Jun Cao, Zhonghua Tao, Biyun Wang

**Affiliations:** Department of Medical Oncology, Fudan University Shanghai Cancer Center, Department of Oncology, Shanghai Medical College, Fudan University, Shanghai, China 200032

## Abstract

Our study aimed to compare the efficacy and safety of nab-paclitaxel plus cisplatin (AP) with nab-paclitaxel plus gemcitabine (AG) in patients with metastatic breast cancer (MBC). We collected data from two single-arm, phase II MBC studies. In NCT01149798, seventy-three MBC patients received 125 mg/m^2^ nab-paclitaxel on days 1, 8 and 15 followed by 75 mg/m^2^ cisplatin on day 1 of a 28-day cycle. In NCT01550848, eighty-four MBC patients received 125 mg/m^2^ nab-paclitaxel and 800 mg/m^2^ gemcitabine on days 1, 8, and 15 of a 28-day cycle. The endpoints were the overall response rate (ORR), progression-free survival (PFS), overall survival (OS) and safety profiles of these regimens. Among the 157 patients included, the ORR were 67.1% and 52.4% for the AP and AG arms, respectively (odds ratio [OR] = 0.246; hazard ratio [HR] = 1.485; 95% confidence interval [CI], 0.762–2.985). After median follow-up periods of 26.3 and 23.3 months in the AP and AG arms, the median PFS were 9.8 months (95%CI, 8.1–11.6) and 8.1 months (95%CI, 6.8–9.4), respectively, while the median OS were 26.9 months (95%CI, 22.4–31.4) and 25.5 months (95%CI, 19.3–31.4), respectively. Neither PFS nor OS adjusted for the number of metastases, occurrence of liver metastasis and chemotherapeutic lines differed significantly between the two arms (PFS:HR = 0.769; 95%CI, 0.541–1.092; p = 0.142; OS:HR = 0.686; 95%CI, 0.426–1.104; p = 0.120). However, PFS was significantly better with AP than with AG in metastatic triple-negative breast cancer (mTNBC) patients (HR = 0.308; 95%CI, 0.129–0.732; p = 0.008). Adverse events were more common with AP than with AG, except for edema and myalgia. Both regimens showed substantial efficacy and were tolerated well in MBC patients. mTNBC who received AP rather than AG showed longer PFS. However, adverse events were more common with AP. Thus, AP may be worth recommending to mTNBC, while AG may be a better alternative for MBC patients with other subtypes.

## Introduction

Breast cancer is the most common malignancy and a main cause of death in women worldwide. Metastatic breast cancer (MBC) is considered incurable, with a median 5-year survival rate of only 26%^1^, reflecting a need for both therapies and insights into the metastatic process. Chemotherapy remains the cornerstone for treating MBC patients with triple-negative, human epidermal growth factor-2 (HER-2)-positive or luminal breast cancer who are resistant to endocrine therapy.

Nanoparticle albumin-bound paclitaxel (nab-paclitaxel) is a novel formulation that does not require polyethylene castor oil as a solvent and reduces both the infusion time and the risk of solvent-induced allergic reactions^2^. Nab-paclitaxel exhibits superior efficacy to solvent-based paclitaxel in terms of a higher response rate (RR), a longer time to progression (TTP) and a trend for longer overall survival (OS)^3^. Additionally, nab-paclitaxel is effective against MBC. The combination of nab-paclitaxel and cisplatin with weekly trastuzumab showed an overall response rate (ORR) of 62.5% and a median progression-free survival (mPFS) of 16.6 months in HER-2-overexpressing MBC^4^. Based on the single-agent activity and synergism of the two drugs in human breast cancer, our center initiated a phase II study (NCT01149798, Registered 24/06/2010) to test the combination of nab-paclitaxel and cisplatin (AP) in MBC patients. The combination regimen showed superior efficacy in terms of mPFS (9.8 months), median overall survival (mOS) (26.9 months) and ORR (67.1%)^5^. The CBCSG006 study, a multicenter randomized phase III trial initiated at our center, assessed the efficacy of a cisplatin + gemcitabine (GP) regimen versus a standard regimen of paclitaxel + gemcitabine (GT) in the first-line treatment of mTNBC. The results of the CBCSG006 study indicated that the platinum-based regimen, GP, could be an alternative or even the preferred first-line chemotherapy for metastatic triple-negative breast cancer (mTNBC)^6^.

The combination of nab-paclitaxel and gemcitabine (AG) was also demonstrated to be an effective regimen in MBC patients. A phase II trial conducted Roy *et al*.^7^ and our center (NCT01550848, Registered 12/03/2012) showed response rates (RRs) of 50% and 52.4% and mPFSs of 7.9 months and 8.1 months in first-line and all-line MBC patients, respectively. The combination demonstrated substantial activity against MBC regardless of the treatment line.

The present study combined the MBC patients from the NCT01149798 and NCT01550848 consecutive phase II trials to investigate which combination was more effective in all-line MBC patients and whether AP is more effective than AG in mTNBC patients.

## Materials and Methods

### Study design and participants

This study combined individual patient data from all patients enrolled in the NCT01149798 and NCT01550848 studies, which were designed independently as prospective single-arm phase II trials to determine the efficacy of each chemotherapy regimen in treating women with MBC.

The inclusion and exclusion criteria for both open-label, phase II, noncomparative studies were similar. Eligible patients were women older than 18 years with pathologically confirmed MBC, a measurable disease according to the Response Evaluation Criteria in Solid Tumors (RECIST) 1.1, adequate organ function, and a life expectancy of at least 3 months. Prior taxanes in adjuvant or neoadjuvant settings were permitted but must have been completed at least 12 months prior to enrollment. Therapy with a taxane as part of metastatic therapy was allowed if treatment was completed at least 3 months before study entry. Patients were excluded if they had clinical evidence of brain metastasis or clinically serious concurrent diseases, pre-existing peripheral neuropathy higher than grade 1, concurrent hormonal therapy or immunotherapy, or other malignancy within the last 5 years that could affect diagnosing or assessing the BC. Patients were also excluded if they had undergone radiotherapy, chemotherapy or treatment with any other drug under investigation within 4 weeks prior to enrollment, did not recover from prior treatment-related toxicity, or were pregnant.

The only difference in the inclusion and exclusion criteria between the two studies was that patients in the NCT01449478 study were required to have an Eastern Cooperative Oncology Group (ECOG) performance status of 0–2, while patients in the NCT01550148 study were required to have an ECOG performance status of 0–1.

Both studies were approved by the Ethics Committee and Institutional Review Board of Fudan University Shanghai Cancer Center. All patients enrolled in the trials provided written informed consent before any study-related procedures. The trials were conducted in accordance with the principles of Good Clinical Practice and the Declaration of Helsinki.

### Procedures

All patients received treatment every 4 weeks. Nab-paclitaxel was administered at 125 mg/m^2^ over 30 minutes on days 1, 8, and 15 without corticosteroid or antihistamine premedication or special infusion sets. In the NCT01449478 study, eligible patients received concurrent cisplatin at 75 mg/m^2^ on day 1 with hydration for 3 continuous days. The treatment continued until disease progression, intolerable toxicity, or a maximum of six cycles^5^. In the NCT01550148 study, eligible patients received concurrent gemcitabine intravenously at a dose of 800 mg/m^2^ over 30 minutes on days 1, 8, and 15. Treatment continued until disease progression, unacceptable toxicity, or patient refusal.

### Assessments

The primary endpoint of both studies and the present study was the ORR, and the secondary endpoints were PFS, OS and safety.

The ORR was defined as the proportion of patients with a measurable disease at baseline who achieved either a complete response (CR) or a partial response (PR). PFS was defined as the time from the date of enrollment to the date of progressive disease or the date of death, and OS was defined as the time interval between study enrollment and death. Patients who remained alive and had not experienced progressive disease were censored at the last clinical visit date. All adverse events (AEs) were graded per the National Cancer Institute Common Terminology Criteria for adverse events, version 4.0.

### Statistical analysis

Patient baseline characteristics between the two studies were compared using the Chi-square test. OS and PFS were estimated using the Kaplan-Meier method and compared using the log-rank test. Hazard ratios (HRs) with two-sided 95% confidence intervals (CIs) were calculated by adjusted Cox proportional hazards models for two arms with subgroup comparisons. For the safety analysis, patients who had received at least one treatment dose were assessed. AEs were compared using the Chi-square test. All statistics were analyzed using SPSS 20.0 software (IBM Corporation, Armonk, NY, USA). P values less than 0.05 were considered significant.

## Results

### Baseline characteristics

Seventy-three women entered the NCT01149798 study between June 2010 and May 2011, and 84 women enrolled in the NCT01550848 study from January 2012 to July 2014. All patients received at least one dose of chemotherapy; thus, 157 patients were included in the present efficacy and safety analysis.

Baseline characteristics of both trials are listed in Table 1. The baseline characteristics were well balanced between both studies, except for the number of metastases, occurrence of liver metastasis and chemotherapeutic lines. The percentages of patients with ≥ 2 metastases and liver metastasis were higher in the AG arm, while more patients experienced ≥ 2 lines of chemotherapy in the AP arm. However, the baseline characteristics in the TNBC subgroup were well balanced between both groups (Supplementary Table 1).

**Table 1:** Baseline characteristics

Patient Characteristic	AP (*n* = 73)	AG (*n* = 84)	p-value
Median age, years (range)	49 (33–65)	50.5 (28–70)	0.647
Median follow-up, months	26.3	23.3	0.472
Amenorrhea			0.581
Premenopausal	29 (39.7)	36 (42.9)	
Postmenopausal	44 (60.3)	48 (57.1)	
Number of metastatic organ sites			0.014*
<2	15 (20.5)	6 (7.1)	
≥2	58 (79.5)	78 (92.9)	
**Metastatic sites**			
Visceral	59 (80.8)	69 (82.1)	0.832
Lung	40 (54.8)	39 (46.4)	0.296
Liver	27 (37.0)	48 (57.1)	0.012*
Non-visceral	14 (19.2)	15 (17.9)	0.832
Subgroups			0.370
Luminal type	46 (63)	61 (72.6)	
HER-2 positive	8 (11)	10 (11.9)	
Triple-negative	16 (21.9)	12 (14.3)	
Unknown	3 (4.1)	1 (1.2)	
Lines of chemotherapy			0.012*
First line	36 (49.3)	59 (70.2)	
Second line	28 (38.4)	15 (17.9)	
Third line or more line	9 (12.3)	10 (11.9)	
**Prior chemotherapy**			
Anthracycline	57 (78.1)	68 (81.0)	0.66
Taxanes	43 (58.1)	55 (65.5)	0.40

Abbreviations: AP, nab-paclitaxel plus cisplatin; AG, nab-paclitaxel plus gemcitabine; HR, hazard ratio; CI, confidence interval; HER-2, human epidermal growth factor receptor-2.

### Efficacy

In the AP arm, 7 patients (9.6%) achieved CRs, while 42 patients (57.5%) achieved PRs, accounting for an ORR of 67.1% for the total population. In the AG arm, the ORR for the total population was 52.4%, with 2 CRs (2.4%) and 42 PRs (50.0%). The difference in ORR was not statistically significant for the total population (OR = 0.246; HR = 1.485; 95%CI, 0.762–2.985).

**Figure 1:**
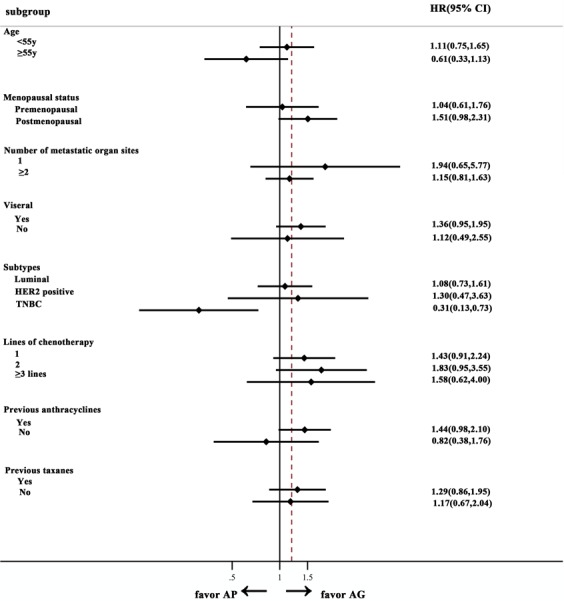
Forest plot of progression-free survival in AP and AG arms. Abbreviations: AP, nab-paclitaxel plus cisplatin; AG, nab-paclitaxel plus gemcitabine; HR, hazard ratio; CI, confidence interval; HER-2, human epidermal growth factor receptor-2; TNBC, triple-negative breast cancer.

The median follow-up period was 26.3 months in the AP arm; 66 patients showed disease progression, and 37 patients died. The median PFS and OS were 9.8 months (95%CI, 8.1–11.6) and 26.9 months (95%CI, 22.4–31.4), respectively. After the median follow-up of 23.3 months with the AG arm, 78 patients experienced progression, and 59 patients died. The mPFS was 8.1 months (95%CI, 6.8–9.4 months), while the mOS was 25.5 months (95%CI, 19.3–31.4). The number of metastases, occurrence of liver metastasis and chemotherapeutic lines were unbalanced between the two groups at baseline, which were potential confounding factors in comparing the variables’ efficacy between the two arms. As such, we conducted an analysis that adjusted the PFS and OS for these predefined covariates for the total population. We found no significant difference for either PFS or OS (PFS: adjusted HR = 0.769, 95%CI, 0.541–1.092; OS: adjusted HR = 0.686, 95%CI, 0.426–1.104) (Table 2). An exploratory analysis confirmed the results’ consistency across all subgroups except the triple-negative subgroup **(**Fig. 1**)**.

**Table 2:** Response rate, progression free survival and overall survival with AP and AG arms.

	AP (n = 73)	AG (n = 84)	HR (95%CI)	p value
**Response**				
Complete response	7 (9.6%)	2 (2.4%)		
Partial response	42 (57.5%)	42 (50.0%)		
Stable disease	12 (16.4%)	22 (26.2%)		
Progressive disease	12 (16.4%)	10 11.9%)		
Missing data or not assessable	0 (0%)	8 (9.5%)		
Overall response rate	49 (67.1%)	44 (52.4%)		0.061
**Progression free survival**				
Number of events	66	78		
Median progression free survival, months (95%CI)	9.8	8.1	0.769 (0.541–1.092)	0.142
**Overall survival**				
Number of events	37 (50.7%)	59 (70.2%)		
Median overall survival, months (95%CI)	26.9	25.5	0.686 (0.426–1.104)	0.120

AP, nab-paclitaxel plus cisplatin; AG, nab-paclitaxel plus gemcitabine; HR, hazard ratio; CI, confidence interval. *Tumor assessment data were missing or not assessable for response because of consent withdrawal or receiving other anticancer drugs before the first assessment in the modified intention-to-treat and per-protocol populations.

In the TNBC subgroup, 11 patients (68.8%) achieved a PR, leading to an ORR of 68.8% in the AP arm. However, in the AG arm, no patients achieved a CR, while 5 patients (46.7%) achieved a PR, accounting for an ORR of 46.7%. However, this difference in ORR was not statistically significant (OR = 0.200; HR = 3.000; 95%CI, 0.560–16.071).

**Figure 2:**
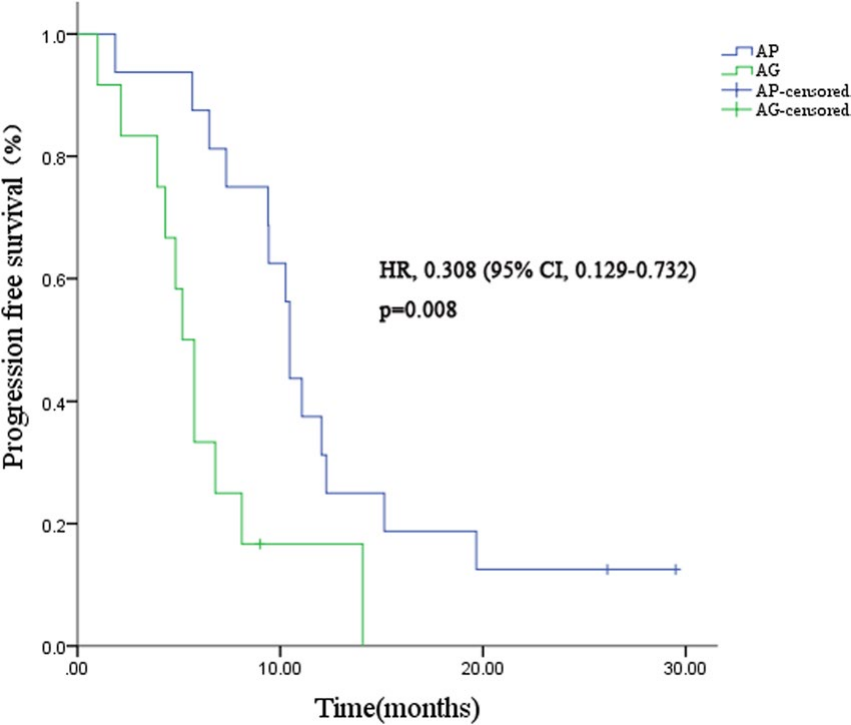
Kaplan–Meier curves for progression-free survival in triple-negative subgroup. Abbreviations: AP, nab-paclitaxel plus cisplatin; AG, nab-paclitaxel plus gemcitabine; HR, hazard ratio; CI, confidence interval.

**Figure 3:**
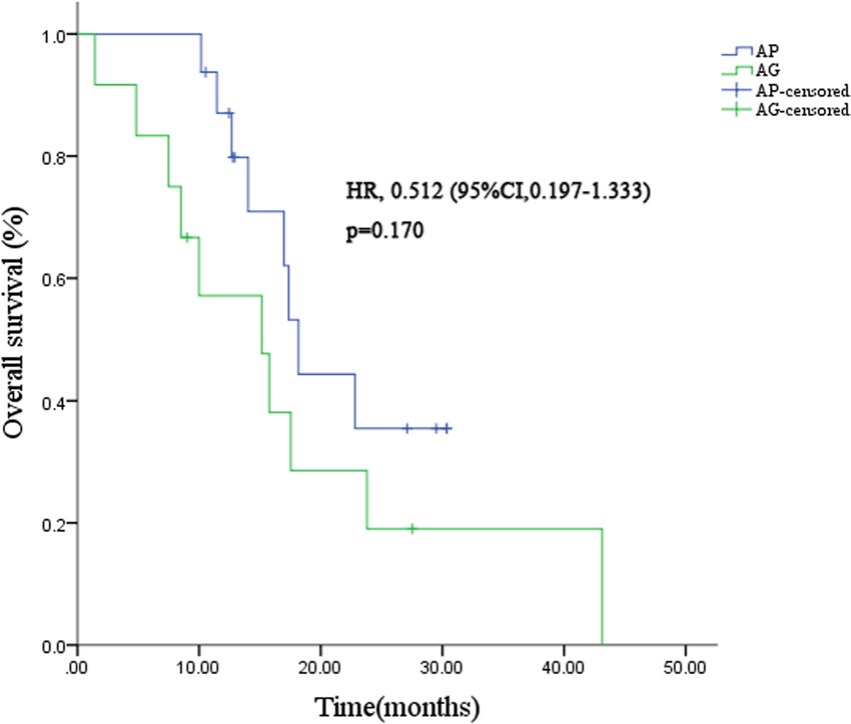
Kaplan–Meier curves for overall survival in triple-negative subgroup. Abbreviations: AP, nab-paclitaxel plus cisplatin; AG, nab-paclitaxel plus gemcitabine; HR, hazard ratio; CI, confidence interval.

The mPFS was significantly longer in patients who received AP than in those who received AG (HR = 0.308; 95%CI, 0.129–0.732; p = 0.008) **(**Fig. 2**)**. A trend towards improved OS was observed in patients who received nab-paclitaxel plus cisplatin, but the difference was not statistically significant (HR = 0.512; 95%CI, 0.197–1.333; p = 0.170) **(**Fig. 3**)**.

### Safety

Patients who received at least one chemotherapy dose were analyzed for safety. In the AP and AG arms, 384 and 360 cycles were administered, with medians of 6 and 5 cycles, respectively. The incidences of drug-related dose reduction were higher for AP than for AG (37.0% vs 10.7%, p = 0.000).

Neutropenia was the most common grade 3 or 4 AE in both studies and was significantly more common in the AP arm (84.9% vs 45.2%, p = 0.000). Except for neutropenia, grades 3 or 4 febrile neutropenia and anemia were more common in the AP arm than in the AG arm (12.3% vs 2.4%, p = 0.03; 17.8% vs 4.8%, p = 0.02; respectively), whereas grades 3 and 4 thrombocytopenia were less common in the AP arm (1.4% vs 8.63%, p = 0.11).

The most common grade 3 or 4 nonhematological AE in both studies was neuropathy, which occurred more frequently in patients who received AP (26% vs 7.1%, p = 0.001). In addition, patients who received AP experienced significantly more all-grade anorexia, fatigue, alopecia, nausea, vomiting, diarrhea, constipation, and abdominal pain but less all-grade edema and myalgia.

No treatment-related deaths were reported in either study.

The drug-related AEs are summarized in Table 3.

**Table 3:** Drug related AEs, Occurring in more than 10% of patients.

AE	AP (n = 73)			AG (n = 84)			p value
	Any grade	Grade 3	Grade 4	Any grade	Grade 3	Grade 4	
**Hematologic**							
Neutropenia	72 (98.6%)	16 (21.9%)	46 (63%)	63 (75.0%)	21 (25.0%)	17 (20.2%)	0.000
Febrile neutropenia	9 (12.3%)	9 (12.3%)	0	2 (2.4%)	1 (1.2%)	1 (1.2%)	0.0338
Anemia	68 (93.2%)	10 (13.7%)	3 (4.1%)	21 (25.0%)	4 (4.8%)	0	0.018
Thrombocytopenia	16 (21.9%)	1 (1.4%)	0	17 (20.2%)	6 (7.1%)	1 (1.2%)	0.069
**Non-Hematologic**							
Neuropathy	53 (72.6%)	19 (26%)	0	42 (50.0%)	6 (7.1%)	0	0.001
Anorexia	24 (33.0%)	0	0	3 (3.6%)	0	0	0.000
Fatigue	25 (34.2%)	0	0	14 (16.7%)	0	0	0.011
Rash	27 (37%)	1 (1.4%)	0	28 (33.3%)	2 (2.4%)	0	0.238
Alopecia	73 (100.0%)	0	0	34 (40.5%)	0	0	0.000
Nausea	47 (64.4%)	2 (2.7%)	0	16 (19.1%)	0	0	0.000
Vomiting	45 (61.6%)	2 (2.7%)	0	14 (16.7%)	0		0.000
Diarrhea	9 (12.3%)	2 (2.7%)	0	2 (2.4%)	0	0	0.0339
Constipation	12 (16.4%)	0	0	0	0	0	0.000
Abdominal pain	10 (13.7%)	0	0	0	0	0	0.000
Skin hyperpigmentation	14 (19.2%)	0	0	8 (9.5%)	0	0	0.082
Blurred vision	4 (5.5%)	0	0	8 (9.5%)	0	0	0.516
Nail change	8 (11.0%)	0	0	19 (22.6%)	0	0	0.053
Edema	2 (2.7%)	0	0	20 (23.8%)	0	0	0.000
Myalgia	6 (8.2%)	0	0	20 (23.8%)	1 (1.2%)	0	0.009

AP, nab-paclitaxel plus cisplatin; AG, nab-paclitaxel plus gemcitabine; AE, adverse event.

## Discussion

In the present analysis, we collected data from two single-arm phase II trials and compared the efficacy and safety of two chemotherapeutic regimens: AP and AG. Our findings showed that these regimens did not significantly differ in terms of PFS, OS or ORR, and the subgroup analysis confirmed their consistency, except in the TNBC subgroup. In the TNBC subgroup, AP was superior to AG in terms of PFS and showed an improvement trend for OS and ORR. However, the improvement in efficacy came at the cost of an increase in toxicity.

Combinations of taxanes with gemcitabine have been shown to be highly active against MBC with manageable toxicity, and AG had high activity and manageable toxicity. A multicenter phase II study evaluated the efficacy of AG in first-line treatment for MBC patients. Fifty women were administered nab-paclitaxel and gemcitabine, which yielded an ORR of 50%, mPFS of 7.9 months and 6-month OS rate of 91%^7^. In addition, the combination of gemcitabine and paclitaxel (GT) has been recommended in the National Comprehensive Cancer Network (NCCN) guidelines as one of the most effective chemotherapeutic regimens for MBC patients pretreated with anthracycline, based on the results of a pivotal global phase III study. In the study, 529 MBC patients were enrolled and randomly assigned to GT or paclitaxel, and GT resulted in a significantly improved OS, longer TTP, and a better RR, thus making GT a reasonable choice for optimal combination therapy^8^. In our present study, AG had an efficacy similar to that of AP for the total population, but with less acceptable toxicity.

In our study, neutropenia and neuropathy were the most common hematological and nonhematological AEs, respectively, and were more frequent in the AP arm. In addition, higher incidences of grades 3 and 4 febrile neutropenia and anemia were also observed in the AP arm. Although the AP and AG tolerability profiles were manageable, AP showed greater toxicity in our study. Comparing our results with the scientific literature identified no new safety concerns, although the AE incidences varied. In a phase II study, 32 HER-2-positive MBC patients were treated with nab-paclitaxel (100 mg/m^2^) with carboplatin (AUC = 2) on days 1, 8, and 15 of a 28-day cycle. Trastuzumab was administered at 2 mg/kg weekly after a loading dose of 4 mg/kg. This combination therapy showed an ORR of 62.5% and a mPFS of 16.6 months. In this clinical trial, only 16 patients (50%) experienced grade 3 or higher neutropenia, 2 patients (6%) experienced grade 3 or 4 anemia, and 1 patient (3%) experienced grade 3 neuropathy^4^. The AEs found in this trial seemed more tolerable than those in our study possibly because most patients in our study had experienced previous metastatic chemotherapy, while all patients in the aforementioned trial had not previously received metastatic chemotherapy.

In Roy’s study^7^, nab-paclitaxel (125 mg/m^2^) and gemcitabine (1000 mg/m^2^) were administered to MBC patients on days 1 and 8 of a 21-day cycle, and the incidences of grade 3 or 4 neutropenia and anemia were 24% and 14%, respectively. Only one patient (2%) had febrile neutropenia, and four patients (8%) had grade 3 neuropathy. The dose intensities of nab-paclitaxel and gemcitabine were 83.3 mg/m^2^/w and 666.7 mg/m^2^/w, respectively. In our study, the dose intensities of nab-paclitaxel and gemcitabine were 93.75 mg/m^2^/w and 600 mg/m^2^/w, respectively. Considering the differences in dose intensity, the higher AE incidence in our study was reasonable. Another phase II study, the tnAcity trial, enrolled 191 patients and randomly assigned them to receive nab-paclitaxel (125 mg/m^2^) + carboplatin (AUC = 2) (nab-p/C), nab-paclitaxel (125 mg/m^2^) + gemcitabine (1000 mg/m^2^) (nab-P/G), or gemcitabine (1000 mg/m^2^) + carboplatin (AUC = 2) (G/C)^9^. This study directly compared the nab-p/C and nab-p/G regimens, which was similar to our study design. The newly updated results, presented at the 2016 San Antonio Breast Cancer Symposium, showed that the occurrence of grade 3 or higher neutropenia was more frequent for nab-P/C than nab-p/G (40.6% vs 33.3%). However, the occurrences of grade 3 or higher anemia and peripheral neuropathy were similar for nab-P/C and nab-p/G (7.8% vs 8.3%, 4.7% vs 5.0%, respectively)^10^. Similarly, the neoadjuvant WSG-ADAPT-TN study directly compared the efficacy and safety of two chemotherapeutic regimens, nab-paclitaxel + gemcitabine and nab-paclitaxel + carboplatin. Three hundred thirty-six TNBC patients were randomly assigned to receive nab-paclitaxel (125 mg/m^2^) + gemcitabine (1000 mg/m^2^) on d1 and 8, q3w or nab-paclitaxel (125 mg/m^2^) + carboplatin (AUC = 2) on d1 and 8, q3w. The results showed that nab-paclitaxel/carboplatin had high efficacy and excellent tolerability, which were superior to those of nab-paclitaxel/gemcitabine for TNBC. The gemcitabine arm was associated with more frequent dose reductions (19.5% vs 44.4%, p < 0.001) and grade 3–4 ALT elevations (11.7% vs 3.3%, p = 0.01)^11^. The safety results of both the tnAcity and WSG-ADAPT-TN studies showed that nab-paclitaxel + carboplatin had better risk/benefit profiles for mTNBC and TNBC patients, respectively. However, in our study, although AP showed superior efficacy with mTNBC, it also resulted in increased toxicity, likely because carboplatin has more favorable toxicity than cisplatin^12^. Moreover, in both studies, nab-paclitaxel + gemcitabine was administered on d1 and 8, q3w, while in our study, this regimen was administered on d1, 8 and 15 on a 28-day cycle. Furthermore, the WSG-ADAPT-TN study was a neoadjuvant study, and the patients enrolled were chemotherapy-naïve, and patients enrolled in the tnAcity study had not previously received metastatic chemotherapy. However, in our study, while patients in both arms had MBC, 50.7% had previously received metastatic chemotherapy, especially in the AP arm, which may have resulted in the cumulative toxicity that showed in the safety comparison.

The present study showed that AG had a better benefit/risk profile than did AP for the entire population, including patients with the luminal or HER-2-positive subtypes, demonstrating that the AG regimen is suitable for treating MBC. In the TNBC subgroup, AP showed better efficacy than AG, confirming a role of platinum in treating mTNBC.

In recent years, increasing studies have demonstrated the efficacy of platinum in neoadjuvant TNBC settings, with conflicting results regarding the long-term survival benefit. Furthermore, platinum’s role in metastatic settings has been gradually highlighted (Table 4). The TBCRC009 trial assessed the efficacy of platinum monotherapy for mTNBC and enrolled 86 mTNBC patients who were treated with first- or second-line cisplatin or carboplatin. The ORR of the platinum monotherapy was 25.6%, indicating that platinum agents are active in mTNBC^12^. A platinum monotherapy regimen was further investigated in the TNT trial, which enrolled 376 patients randomized to receive carboplatin or docetaxel as a first-line treatment. The TNT trial results showed that carboplatin and docetaxel as first-line treatments in patients with mTNBC had similar efficacies in terms of ORR (31.4% vs 35.6%, respectively) and PFS (3.1 vs 4.5 months, respectively)^13^. These results indicate that single-agent platinum has limited efficacy in treating mTNBC. To further improve its efficacy, several studies have also investigated platinum-based combination chemotherapy in mTNBC. The CBCSG006 study compared the GP and GT regimens in the mTNBC setting, in which GP significantly reduced the HR for PFS (HR = 0.69; 95%CI, 0.52–0.92; p noninferiority < 0.0001, p superiority = 0.009), demonstrating that GP was both noninferior and superior to GT^6^. To further evaluate the platinum-containing regimen for first-line treatment of mTNBC, two ongoing phase II studies were initiated at our center to directly compare the efficacy and safety of cisplatin + nab-paclitaxel or gemcitabine (NCT02546934) and gemcitabine + cisplatin or carboplatin (NCT02341911). We believe that the results of these two studies will provide additional evidence for the superiority of combination regimens with platinum in mTNBC. Additionally, the newly updated results of the tnAcity trial showed the superiority of the combination of nab-paclitaxel and carboplatin. Therefore, nab-paclitaxel + carboplatin is superior to nab-paclitaxel + gemcitabine in mTNBC.

**Table 4:** Platinum-containing regimens for metastatic triple-negative breast cancer.

Study	Regimen	Study design and setting	Number of patients	ORR	mPFS (months)	mOS (months)
Isakoff SJ *et al*.^12^	Cisplatin 75 mg/m^2^ or Carboplatin (AUC = 6)	Phase II, first-or second-line	86	25.6%	2.9	11
Tutt A *et al*.^13^	Carboplatin (AUC = 6), q3w	Phase III, first-line	188	31.4%	3.1	NR
	docetaxel 100 mg/m^2^, q3w		188	35.6%	4.5	NR
HU XC *et al*.^6^ (our center)	Cisplatin,75 mg/m2, d1 + gemcitabine,1250 mg/m^2^, d1,8, q3w	Phase III, first-line	120	64.0%	7.73	NR
	Nab-paclitaxel 175 mg/m^2^, d1 + gemcitabine d1,8, q3w		120	49.0%	6.47	NR
Yardley D A *et al*.^10^	Nab-paclitaxel 125 mg/m^2^ + carboplatin AUC = 2, d1, 8, q3w	Phase II, first-line	64	71.9%	7.4	16.4
	Nab-paclitaxel 125 mg/m^2^ + gemcitabine, 1000 mg/m2, day1, 8, q3w		61	37.7%	5.4	11.9
	Gemcitabine, 1000 mg/m^2^ +carboplatin AUC = 2, days 1, 8, q3w		66	43.9%	6.0	13.7
Fan Y *et al*.^14^	Docetaxel, 75 mg/m^2^ + cisplatin, 75 mg/m^2^, d1, q3w	Phase II, first-line	27	63.0%	10.9	32.8
	Docetaxel, 75 mg/m^2^ + capecitabine bid 2 weeks on, 1 week off, q3w		26	15.4%	4.8	21.5
Zhang J *et al*.^17^ (our center)	Vinorelbine,30 mg/m^2^ + Oxaliplatin,90 mg/m^2^, biweekly, q4w	Phase II, second- or third-line	44	31.6%	4.3	12.6
Li Q *et al*.^18^	Capecitabine,2000 mg/m^2^, d1–14 + cisplatin, 75 mg/m^2^, d1, q3w	Phase II, all lines	33	63.6%	8.2	17.8

ORR, overall response rate; mPFS, median progression free survival; mOS, median overall survival; q3w, every 3 week.

In addition to CBCSG006 and tnAcity, other studies have explored platinum-containing combination regimens in later treatment lines in metastatic settings. A randomized phase II study evaluated the efficacy of docetaxel-cisplatin (TP) and docetaxel-capecitabine (TX) for first-line treatment of mTNBC. Fifty-three patients were enrolled, and the results showed that TP was superior to TX in terms of ORR (63% vs 15.4%, p = 0.001), mPFS (10.9 vs 4.8 months, p < 0.001), and mOS (32.8 vs 21.5 months, p = 0.027)^14^. The vinorelbine and cisplatin combination, an effective regimen in MBC, has also been investigated in mTNBC^15,16^. The efficacy and safety of administering the combination of vinorelbine and oxaliplatin biweekly in the second- or third-line treatment of mTNBC has also been explored in our center. For the 44 patients enrolled, the ORR was 81.6%, and the mPFS and mOS were 4.3 months (95%CI, 3.6–5.0) and 12.6 months (95%CI, 8.1–17.0), respectively^17^. In addition, a phase II study showed that the combination of capecitabine and cisplatin in mTNBC patients pretreated with anthracyclines and taxanes achieved an ORR of 63.6%, with an mPFS and mOS of 8.2 months (95%CI: 4.8–11.6) and 17.8 months (95%CI: 14.4–21.2), respectively^18^.

These clinical studies all provided some level of evidence for platinum activity against mTNBC. Consistent with these results, the platinum-based regimen, AP, in our study, led to significantly longer PFS than the AG regimen. The newly updated results of the tnAcity trial and WSG-ADAPT-TN study further support our findings.

Our study had several limitations. First, our study was not randomized. We combined individual patient data from patients enrolled in two trials; thus, the baseline characteristics may lack equivalency. For example, more patients had liver metastasis in the AG arm, while more patients received 2 or more lines of chemotherapy in the AP arm. Although we used adjusted Cox proportional hazards models to predefine covariates, the statistical power of our results may have been reduced. Second, due to the nature of the study and restricted funds, both studies had limited sample sizes. Thus, these were inadequate and small patient populations. Third, the patients all came from a single center, which may have led to selection bias.

## Conclusions

Although this study was not randomized, but rather a report of two consecutive single-arm studies, our results showed that AP and AG had similar efficacy for the study population; however, in the TNBC subgroup, AP was more effective, although with increased toxicity. Considering the inadequate and small-sized patient population, the results give some suggestions that platinum might play an important role in the treatment of mTNBC, and AP may be worth recommending to mTNBC patients, while AG may be a better alternative for MBC patients with other subtypes. Randomized control trials are still needed to further confirm our results.

## Supplementary information

supplementary information

## Data Availability

The datasets generated and analyzed during the current study are not publicly available due to hospital policy but are available from the corresponding author on reasonable request.

## References

[1] Siegel RL, Miller KD, Jemal A (2016). Cancer statistics, 2016. CA Cancer J Clin..

[2] Ibrahim NK (2002). Phase I and pharmacokinetic study of ABI-007, a Cremophor-free, protein-stabilized, nanoparticle formulation of paclitaxel. Clin Cancer Res..

[3] Gradishar WJ (2005). Phase III trial of nanoparticle albumin-bound paclitaxel compared with polyethylated castor oil-based paclitaxel in women with breast cancer. J Clin Oncol..

[4] Conlin AK (2010). Phase II trial of weekly nanoparticle albumin-bound paclitaxel with carboplatin and trastuzumab as first-line therapy for women with HER2-overexpressing metastatic breast cancer. Clin Breast Cancer..

[5] Sun S (2014). Cisplatin improves antitumor activity of weekly nab-paclitaxel in patients with metastatic breast cancer. Int J Nanomedicine..

[6] Hu XC (2015). Cisplatin plus gemcitabine versus paclitaxel plus gemcitabine as first-line therapy for metastatic triple-negative breast cancer (CBCSG006): a randomised, open-label, multicentre, phase 3 trial. Lancet Oncol..

[7] Roy V (2009). Phase II trial of weekly nab (nanoparticle albumin-bound)-paclitaxel (nab-paclitaxel) (Abraxane) in combination with gemcitabine in patients with metastatic breast cancer (N0531). Ann Oncol..

[8] Albain KS (2008). Gemcitabine plus Paclitaxel versus Paclitaxel monotherapy in patients with metastatic breast cancer and prior anthracycline treatment. J Clin Oncol..

[9] Yardley DA (2015). Phase II/III weekly nab-paclitaxel plus gemcitabine or carboplatin versus gemcitabine/carboplatin as first-line treatment of patients with metastatic triple-negative breast cancer (the tnAcity study): study protocol for a randomized controlled trial. Trials..

[10] Yardley, D. *et al*. Nab-paclitaxel plus carboplatin or gemcitabine vs gemcitabine/carboplatin as first-line treatment for patients with triple-negative metastatic breast cancer: Results from the randomized phase 2 portion of the tnAcity trial. *Cancer Res*. **77** (2017). 10.1093/annonc/mdy201 PMC6096741 29878040

[11] Gluz, O. *et al*. Comparison of Neoadjuvant Nab-Paclitaxel + Carboplatin vs Nab-Paclitaxel + Gemcitabine in Triple-Negative Breast Cancer: Randomized WSG-ADAPT-TN Trial Results. *J Natl Cancer Inst* (2017). 10.1093/jnci/djx258 29228315

[12] Isakoff SJ (2015). TBCRC009: A Multicenter Phase II Clinical Trial of Platinum Monotherapy With Biomarker Assessment in Metastatic Triple-Negative Breast Cancer. J Clin Oncol..

[13] Tutt, A. *et al*. The TNT trial: A randomized phase III trial of carboplatin (C) compared with docetaxel (D) for patients with metastatic or recurrent locally advanced triple negative or BRCA1/2 breast cancer (CRUK/07/012). *Cancer Res*. **75** (2015).

[14] Fan Y (2013). Docetaxel-cisplatin might be superior to docetaxel-capecitabine in the first-line treatment of metastatic triple-negative breast cancer. Ann Oncol..

[15] Vassilomanolakis M (2000). Vinorelbine and cisplatin in metastatic breast cancer patients previously treated with anthracyclines. Ann Oncol..

[16] Vassilomanolakis M (2003). Vinorelbine and cisplatin for metastatic breast cancer: a salvage regimen in patients progressing after docetaxel and anthracycline treatment. Cancer Invest..

[17] Zhang J (2015). A phase II trial of biweekly vinorelbine and oxaliplatin in second- or third-line metastatic triple-negative breast cancer. Cancer Biol Ther..

[18] Li Q (2015). A phase II study of capecitabine plus cisplatin in metastatic triple-negative breast cancer patients pretreated with anthracyclines and taxanes. Cancer Biol Ther..

