# Supplementary material for: Cisplatin shows greater efficacy than gemcitabine when combined with nab-paclitaxel in metastatic triple-negative breast cancer
Source: Sci Rep. 2019 Mar 5;9:3563. doi: 10.1038/s41598-019-39314-y (PMC6400896; doi:10.1038/s41598-019-39314-y)
Supplement: Supplementary file 1 — supplementary information [file 41598_2019_39314_MOESM1_ESM.pdf]

Cisplatin shows greater efficacy than gemcitabine when combined with nab-paclitaxel in metastatic triple-negative breast cancer

Yi Li[1]#, Yannan Zhao [1]#, Chengcheng Gong[1], Yizhao Xie[1], Xichun Hu[1], Jian Zhang[1], Leiping Wang[1], Sheng Zhang[1], Jun Cao[1], Zhonghua Tao[1], Biyun Wang[1]

[1]Department of Medical Oncology, Fudan University Shanghai Cancer Center, Department of Oncology, Shanghai Medical College, Fudan University, Shanghai, China 200032

Correspondence to: E-mail: wangbiyun0107@hotmail.com
# These authors contributed equally to this work.

Supplementary Table
Table1. Baseline triple- negative subgroup patient characteristics

Patient Characteristic	AP [[[n=16)	AG (n=12)	p value
Median age, years(range)	49(33-65)	50.5(28-70)	0.647
Median follow up, months	26.3	23.3	0.472
Amenorrhea			0.718
Premenopausal	8(50.0%)	5 (41.7)	
Postmenopausal	8(50.0%)	7 (58.3)	
Number of metastatic organ sites			0.492
<2	2(12.5)	0 (0)	
≥2	14(87.5)	12(100.0)	
Metastatic sites			
Visceral	13(81.3)	10 (83.3)	1.000
Lung	11(68.8)	9(75.0)	1.000
Liver	3(18.8)	5(41.7)	0.231
Non-visceral	3(18.8)	2 (16.7)	0.832
Lines of chemotherapy			0.717
First line	10(62.5)	8(66.7)	
Second line	3(18.8)	1(8.3)	
Third line or more line	3(18.8)	3(25.0)	
Prior chemotherapy			
Anthracycline	12(75.0)	10(83.3)	0.673
Taxanes	12(75.0)	8 (66.7)	0.691

Abbreviations: AP, nab-paclitaxel plus cisplatin; AG, nab-paclitaxel plus gemcitabine; HR, hazard ratio; CI, confidence interval; HER-2, human epidermal growth factor receptor-2

Protocol of NCT01149798

A Phase II study of weekly nab-paclitaxel in combination with cisplatin in patients with

metastatic breast cancer

**Site：**	Department of Medical Oncology, Fudan University Shanghai Cancer Center
**Principal Investigator:**	Xi-Chun Hu, MD, Bi-yun Wang, MD
**Tel：**	13701748410
**Version:**	1.1
**Date：**	Jun 2011

SUMMARY

**Study design**	Prospective、single-site、open-label phase II clinical study
**Primary end point**	Objective Response Rate, ORR
**Secondary end points**	Progression Free Survival, PFS<br>Safety<br>Overall Survival, OS
**patient characteristics**	73 locally advanced or metastatic breast cancer patients
**Inclusion Criteria**	1. Females with age ≥18 years old；<br>2. Histologically identified locally advanced (unresectable) or metastatic breast cancer；<br>3. ECOG 0-2；<br>4. Normal functions with liver, renal and bone marrow；<br>5. Life expectancy greater than 12 weeks；<br>6. No severe medical history of heart, lung, liver, renal and so on;<br>7. ICF (Informed Consent Form) before enrollment；<br>8. Good compliance.
**Treatment**	ABX (Abraxane) 125 mg/m2 ， intravenous infusion ，<br>30min，day 1, 8, 15,q4w<br>DDP (Cisplatin) 75 mg/m2，intravenous infusion，<br>120 min，day 1, 2, 3,q4w<br>(DDP must be hydrated for 3 days Per cycle)<br>Until disease progression or intolerable toxicity.
**Assessment of efficacy**	According to RECIST 1.1
**Assessment of AE**	According to CTCAE 4.0

1. BACKGROUND AND THEORETICAL FOUNDATION

Metastatic breast cancer (MBC) is known as an incurable disease with a median 5-year overall survival about 24-36 months and median 5-year survival rate around 23.8-30% [1]. Cytotoxic chemotherapy remains as the base stone in the treatment of MBC. The main aim of MBC treatment study is to improve efficacy and alleviate the toxicities.

Taxane is an effective anti-cancer drug that is widely used to treat patients with breast cancer [2]. As it is hydrophobic, taxane is formulated with the micelle-forming vehicle Cremophor EL (CrEL) or Tween 80 to increase its drug solubility. High doses of steroids and antihistamines must be administered as premedication to prevent hypersensitivity. In addition, paclitaxel requires a non-polyvinyl chloride container and is administered via a special infusion tubing to prevent CrEL to leach plasticizers. In-line filters call for three hours' infusion time [2].

Abraxane is a CrEL-free, protein-stabilized, nanoparticle albumin-bound paclitaxel (nab-paclitaxel) formulation. It was approved by U.S. Food and Drug Administration (FDA) in 2004, and by China SFDA/CDE on 2008 to treat metastatic breast cancer with previous chemotherapy history. Paclitaxel was wrapped in 130nm nanoparticle albumin to become soluble. No CrEL was used, so patients need no premedication. It could be safely infused in 30 minutes with normal infusion tubing at a high dose of 260 mg/m^2 every 3 weeks, which is 50% higher than standard paclitaxel. In addition, albumin is the carrier of the nutrition to cells, previous studies suggested albumin accumulate in rapidly growing cancer cells. Nab-paclitaxel has an albumin delivery system that increases drug targeting to the tumor cell [4-5]. In a phase III trial, nab-paclitaxel showed a significantly higher overall response rate (ORR), a longer time to progression (TTP), and greater overall survival (OS) in patients treated with second-line or greater therapy compared with patients who received sb-paclitaxel. Furthermore, weekly nab-paclitaxel can be safely administered at doses exceeding those typically used for sb-paclitaxel, is effective in taxane resistant patients, and as a single drug is more effective than docetaxel, which was previously considered the most potent drug for MBC. Therefore, weekly nab-paclitaxel is becoming a standard of care for MBC patients.

To further improve the efficacy of weekly nab-paclitaxel in MBC patients, several studies tested combinations by adding other chemotherapeutic agents or biologicals. Therefore, we designed the current Phase II study to assess the efficacy and safety of combination of nab-

paclitaxel and cisplatin in patients with MBC.

2. STUDY OBJECTIVES

2.1 Primary Objectives

Objective Response Rate (ORR): ORR was defined as the percentage of patients who achieved complete response (CR) and partial response (PR) by Response Evaluation Criteria in Solid Tumors (RECIST) version 1.1 criteria.

2.2 Secondary Objectives

1. PFS (Progression Free Survival)： PFS was defined as the time from enrollment to the first documented disease progression or death from any cause.

2. Safety: AEs were evaluated, graded and recorded according to National Cancer Institute Common Terminology Criteria for Adverse Events (CTCAE), version 4.0.

3. Overall survival (OS): OS was defined as the time from enrollment to the date of death from any cause or last follow-up.

3. STUDY PLAN

This is a prospective、single-site、open-label phase II clinical study. 73enrolled patients will receive abraxane and cisplatin until disease progression, intolerable toxicity or a maximum of six cycles.

Patients will receive treatment assessment every 2 cycles of treatment until disease progression according to RECIST1.1.

4. SELECTION OF SUBJECT POPULATION

4.1 Inclusion Criteria

1. Females with age ≥18 years old；
2. Histologically identified locally advanced (unresectable) or metastatic breast cancer；
3. ECOG 0-2；
4. Adequate functions with liver, renal and bone marrow:
5. Hemoglobin (Hb) ≥90g/L, white blood cell (WBC) ≥3.5×109/L, absolute neutrophil count (ANC) ≥1.5×109/L, platelet (PLT)<100×109/L;
6. Serum bilirubin＜upper limit of normal (UNL);
7. Without liver metastasis, aspartate aminotransferase/serum glutamic oxalacetic transaminase (AST/SGOT)， serum alanine transaminase/serum glutamic pyruvic transaminase (ALT/SGPT) and alkaline phosphatase (ALP) ≤2.5×UNL;
8. With liver metastasis, AST/SGOT, ALT/SGPT and ALP >5×UNL;
   Serum creatinine ≤ 1.5×UNL;
9. Life expectancy greater than 12 weeks；
10. No severe medical history of heart, lung, liver, renal and so on;
11. ICF (Informed Consent Form) before enrollment；
12. Good compliance.

4.2 Exclusion Criteria

1. Pregnancy or breast feeding (In women at childbearing age, HCG must be examined in 14 days prior to the first administration to exclude pregnancy. If positive, ultrasound examination is necessary to exclude pregnancy);

2. Women at childbearing age who refuse contraception measures in study;

3. Treatment with radiotherapy at the axial skeleton within 4 weeks before the first treatment or has not recovered from all toxicities of previous radiotherapy;

4. Use of other clinical trial medication or participating in other clinical trial currently or within the last 4 weeks.

5. Uncontrolled brain metastases. Patients with brain metastases must be locally treated and the disease must be stable for at least one month at the time of enrolling;

Participation in any investigational drug study within 4 weeks preceding treatment start;

6. Concurrent other malignancy at other sites or previous other cancer within the last 5 years, with the exception of adequately treated in situ carcinoma of cervix uteri or basal or squamous cell carcinoma of the skin or a contralateral breast cancer;

7. Patients having a history of clinically significant cardiovascular, hepatic, respiratory or renal diseases, clinically significant hematological and endocrinal abnormalities, clinically significant neurological or psychiatric conditions

8. Serious uncontrolled infections.

5. TREATMET

ABX (Abraxane) 125 mg/m2，intravenous infusion，30min，day 1, 8, 15, q4w,

DDP (Cisplatin) 75 mg/m2， intravenous infusion， 120 min， day 1, 2,3，q4w,

until disease progression, intolerable toxicity or a maximum of six cycles.

6. CONCOMITANT MEDICATIONS

6.1 Premedication

5-Hydroxytryptamine-3 Receptors Antagonist, dexamethasone or promethazine can be used to prevent and relieve vomiting after chemotherapy according to doctors' decision.

6.2 Supporting treatment

All patients can receive standard supporting treatment, including blood transfusion or anti-biotics.

6.2.1 Colony-stimulating factor

Granulocyte colony-stimulating factor (G-CSF) can be used for neutropenia, but not as a preventive medication.

6.2.2 Platelet transfusion

When grade II or high platelet count decrease appears, platelet transfusion, IL-11 or thrombopoietin.

6.2.3. Bisphosphonates

Bisphosphonates for bone metastases confirmed by radiological evidence or ECT at baseline are allowed. Use of bisphosphonates after clinical trial entry with no bone metastases evidence at baseline is considered as disease progression.

6.2.4. Other concomitant medications

Other concomitant medications should be minimized. But when it comes to the interests of the patients and does not interfere with the study, it can be determined by the principal investigators. All concomitant medications used within 4 weeks before first administration should be recorded in the case report form.

Following medication is not recommended to use, except that it is necessary to use in patients:

- Medications with potential inducing effects of CYP2C8 and CYP3A4: rifampicin, carbamazepine, diphenylhydantoin sodium, efavirenz；
- Medications with potential depressing effects of CYP2C8 and CYP3A4: erythromycin, fluoxetine, gemfibrozil;

7. DOSE MODIFICATION AND TREATMENT DELAY

7.1 Treatment of adverse events and principal of dose modification

If ANC$\leq$1.5 x 10^9/L and PLT<100 x10^9/L on day1 of every cycle of chemotherapy, the chemotherapy should be delayed. Treatment should be interrupted for non-hematological toxicities of any grade II above (except alopecia, nausea and vomiting) until it recovers to grade II or less. The duration of chemotherapy delay is permitted up to 14 days to recover from hematological and non-hematological toxicities. Dose reduction is permitted up to twice in all courses. Dose reduction should be based on the most severe toxicity of the previous course. The first dose is lowered by 20%-25% and the second by 20%-25%, so that the dose can be lowered to a maximum of 50%-60% of the preset dose. Those who were still intolerant after two doses were discontinued. The drug was reduced according to the toxicity of the previous course of treatment.

7.2 Reduction of both two drugs

1. Grade III/IV febrile neutropenia occur(with a single temperature of >38.3℃ or a sustained temperature of ≥38 ℃ for more than one hour)；
2. Grade IV neutropenia for more than 7 days;
3. Other situations that investigators decide to reduce the dose;

7.3 Reduction of Abraxane

4. If grade II or above neurologic toxicity occurs, patients should take the

electromyogram to confirm and receive symptomatic treatments. Treatment should be interrupted for 14 days at most until it recovers to grade II or less, and dose should be reduced according to principal of dose modification.

5. Except alopecia, nausea and vomiting, other severe non-hematological toxicities, which are considered related to Abraxane, doctors can reduce the dose of Abraxane according to principal of dose modification.

7.4 Reduction of Cisplatin

6. The dosage of DDP was adjusted according to renal function. When 45ml/min ≤ creatinine clearance rate (CCI) < 60ml / min, DDP decreases by 25%; when 30ml/min ≤ creatinine clearance rate (CCI) < 45ml / min, DDP decreases by 50%; DDP reduction should be suspended when creatinine clearance (CCI) < 30 ml / min.

8. TREATMENT DISCONTINUATION

Patients will discontinue the study treatment and follow-up until death under the following circumstances:

1. Disease progression during the study;
2. Patients who are still intolerable after twice dose modification;
3. A maximum of six cycles
4. Treatment are interrupted for more than 2 weeks;
5. Pregnancy;
6. Protocol violations that render the patient unsuitable for further treatment;
7. Any other reason deemed appropriate by the investigator.

9. ESTIMATION OF SAMPLE SIZE

The ORR of combination was hypothesized as 60 % with the addition of cisplatin in the whole population compared with 40 % for weekly nab-paclitaxel monotherapy, as reported in previous studies with 80 % power, type 1 error, α=0.05. 72 patients were required in consideration of 10 % expulsion rate.

10. MEASUREMENTS OF STUDY VARIABLES

10.1 Tumor assessment

Radiographic assessment (CT or MRI) and tumor markers for disease evaluation should be performed within 4 weeks prior to the treatment, with QOL questionnaire FACT-G+B+TAXANE (version 4). RECIST 1.1 is used to evaluate efficacy, including measurable and unmeasurable lesions, target or non-target lesions. Objective tumor assessment is evaluated as complete response (CR), partial response (PR), stable disease (SD) and disease progression (PD).

Baseline assessment should be performed within 4 weeks prior to the treatment, and as near as possible. Radiographic assessment includes chest, abdomen, pelvis CT or MRI. Measurement of tumor assessment in follow-up visits should be the same with the baseline. Other suspected lesions may be evaluated at baseline and follow-up visits. After baseline assessment, all enrolled patients should receive the response assessment and QOL questionnaire every two cycle ± 3 days, until disease progression occurs according to RECIST 1.1. If patients receive a response assessment not in pre-set time, next response assessment should be administered as the pre-set time, as long as the patients still enroll in this study. Efficacy of every patients who reach ORR (CR+PR) should be confirmed after at least 4 weeks. Interval of next response assessment should be 2 courses of treatment.

If the patient withdraws the study before disease progression (and/or receive other

treatment besides the study), the patient should be followed up until disease progression according to RECIST 1.1.

Objective tumor assessment is evaluated as complete response (CR), partial response (PR), stable disease (SD) and disease progression (PD) according to RECIST 1.1. Evaluation of target lesion as PD should be at least a 20% increase in the sum of diameters, taking as reference the smallest sum on study. Except PD, other response (CR, PR and SD) should be compared with the baseline.

If the investigator and clinical research coordinator cannot confirm progression, especially response of non-target lesion and emerging of new lesion, the treatment can continue until next evaluation, or a quick re-evaluation dependent on clinical practice. If disease progression is confirmed in re-evaluation, the date of disease progression should be the time that response is first suspected as PD.

If disease progression is dependent on the progression of non-target disease, there must be an overall level of substantial worsening in non-target disease such that, even in presence of SD or PR in target disease, the overall tumor burden has increased sufficiently to merit discontinuation of therapy. A modest 'increase' in the size of one or more non-target lesions is usually not sufficient to quality for unequivocal progression status.

After disease progression, follow-up visits should be every 3 months until death of patients.

10.2 Evaluation of adverse event

Adverse events will be collected after every cycle of chemotherapy, according to the physical examination and laboratory assessment. Adverse events were graded using the National Cancer Institute Common Terminology Criteria for Adverse Events version 4.0.

11. ADVERSE EVENT

11.1 Definitions

An adverse event is the development of an undesirable medical condition or the deterioration of a pre-existing medical condition following or during exposure to a

pharmaceutical product, whether or not considered causally related to the treatment.

11.2 Evaluation of Adverse Event

Adverse Events will be collected throughout the treatment period. Every adverse event should be documented in the CRF, whether or not considered causally related to the treatment.

11.3 Definition of Serious Adverse Event (SAE)

A serious adverse event is an AE occurring during any study phase and fulfils one or more of the following criteria:

- results in death
- is immediately life-threatening
- requires in-patient hospitalization or prolongation of existing hospitalization
- results in persistent or significant disability or incapacity
- is a congenital abnormality or birth defect

Adverse Event is an important medical event that may jeopardize the subject or may require medical intervention to prevent one of the outcomes listed above. If any SAE occurs in the course of the study, then Investigators or other site personnel should inform immediately.

11.4 Grading of Adverse Event

Adverse event is graded as Grade 1-4 by National Cancer Institute Common Terminology Criteria for Adverse Events version 4.0. If there is no grading according CTCAE 4.0, adverse event can be evaluated as follows:

Mild: mild symptoms; intervention not indicated; no influence on ADL;

Moderate: moderate symptoms; minimal, local or noninvasive intervention indicated; no influence on ADL;

Severe: medically significant but not immediately life-threatening; limiting ADL;

Life-threatening consequences: life-threatening and need urgent intervention; or death

related to AE.

11.5 Reporting of Adverse Event

The following variables will be collected for each AE: AE (verbatim), the date when the AE started and stopped, intensity, whether the AE is serious or not, action taken with regard and outcome. Investigators or other site personnel report as requirements of SFDA. In this study, SAE should be reported to principal investigator, GCP, ethics immediately but no later than 24 hours.

12. REFERENCE

1. Jemal A.Cancer Statistics, 2007. CA Cancer J Clin, 2007, 57: 43-66.
2. Van Zuylen.Role of formulation vehicles in taxane pharmacology. Investigational New Drugs. 2001 May;19(2):125-41.
3. Ibrahim. Phase I and Pharmacokinetic Study of ABI-007, a Cremophor-free, Protein-stabilized, Nanoparticle Formulation of Paclitaxel.Clinical Cancer Research. *Vol. 8, 1038–1044, May 2002*
4. Desai, N., V. Trieu, et al. Increased antitumor activity, intratumor paclitaxel concentrations, and endothelial cell transport of cremophor-free, albumin-bound paclitaxel, ABI-007, compared with cremophor-based paclitaxel. Clin Cancer Res **12**(4): 1317-24.
5. Henderson, I. C. and V. Bhatia."Nab-paclitaxel for breast cancer: a new formulation with an improved safety profile and greater efficacy." Expert Rev Anticancer Ther 2007 **7**(7): 919-43.

A Phase II study of weekly nab-paclitaxel in combination with gemcitabine in patients with metastatic breast cancer

**Site：**	Department of Medical Oncology, Fudan University Shanghai Cancer Center
**Principal Investigator:**	Xi-Chun Hu, MD, Bi-yun Wang, MD
**Tel ：**	13701748410
**Version:**	1.1
**Date：**	Nov 2011

SUMMARY

**Study design**	Prospective、single-site、open-label phase II clinical study
**Primary end point**	Objective Response Rate, ORR
**Secondary end points**	Progression Free Survival, PFS<br>Safety<br>Overall Survival, OS
**patient characteristics**	84 locally advanced or metastatic breast cancer patients
**Inclusion Criteria**	1. Females with age ≥18 years old；<br>2. Histologically identified locally advanced (unresectable) or metastatic breast cancer；<br>3. ECOG ≤1；<br>4. Normal functions with liver, renal and bone marrow；<br>5. Life expectancy greater than 12 weeks；<br>6. No severe medical history of heart, lung, liver, renal and so on;<br>7. ICF (Informed Consent Form) before enrollment；<br>8. Good compliance.
**Treatment**	ABX (Abraxane) 125 mg/m2, intravenous infusion, 30min, day 1,8,15, q4w<br><br>GEM (Gemcitabine) 800 mg/m2，intravenous infusion，30min，day 1, 8, 15,q4w<br>Until disease progression or intolerable toxicity.
**Assessment of efficacy**	According to RECIST 1.1
**Assessment of AE**	According to CTCAE 4.0

1. BACKGROUND AND THEORETICAL FOUNDATION

Metastatic breast cancer (MBC) is known as an incurable disease with a median 5-year overall survival about 24-36 months and median 5-year survival rate around 23.8-30%[1]. Cytotoxic chemotherapy remains as the base stone in the treatment of MBC. The main aim of MBC treatment study is to improve efficacy and alleviate the toxicities.

Taxane is an effective anti-cancer drug that is widely used to treat patients with breast cancer[2]. As it is hydrophobic, taxane is formulated with the micelle-forming vehicle Cremophor EL (CrEL) or Tween 80 to increase its drug solubility. High doses of steroids and antihistamines must be administered as premedication to prevent hypersensitivity. In addition, paclitaxel requires a non-polyvinyl chloride container and is administered via a special infusion tubing to prevent CrEL to leach plasticizers. In-line filters call for three hours' infusion time[2].

Abraxane is a CrEL-free, protein-stabilized, nanoparticle albumin-bound paclitaxel (nab-paclitaxel) formulation[3]. It was approved by U.S. Food and Drug Administration (FDA) in 2004, and by China SFDA/CDE on 2008 to treat metastatic breast cancer with previous chemotherapy. Paclitaxel was wrapped in 130nm nanoparticle albumin to become soluble. No CrEL was used, so patients need no premedication. It could be safely infused in 30 minutes with normal infusion tubing at a high dose of 260 mg/m^2 every 3 weeks, which is 50% higher than standard paclitaxel. In addition, albumin is the carrier of the nutrition to cells, previous studies suggested albumin accumulate in rapidly growing cancer cells. Nab-paclitaxel has an albumin delivery system that increases drug targeting to the tumor cell[4, 5].

Roy et al.[6] conducted a phase II trial in US evaluated nab-paclitaxel plus gemcitabine in MBC patients as a first-line treatment. ORR was 50% (complete remission [CR] 8%, partial response [PR] 42%) and the median PFS was 7.9 months. The regimen was well tolerated with incidences of grade 3 and 4 neutropenia 42% and 12% respectively.

In another phase II trial in Europe, nab-paclitaxel (150mg/m^2), gemcitabine (1500mg/m^2) and bevacizumab (10mg/kg) were administered in TNBC patients as first-line therapy on day 1 and 15 of a 28-day cycle. Thirty patients were enrolled with ORR 75.9% and median PFS 10.4 months. Besides, the regimen was well-tolerated[7]. Weekly nab-paclitaxel combined with gemcitabine may appear to be noble combination therapy in MBC patients.

2. STUDY OBJECTIVES

2.1 Primary Objectives

Objective Response Rate (ORR): ORR was defined as the percentage of patients who achieved complete response (CR) and partial response (PR) by Response Evaluation Criteria in Solid Tumors (RECIST) version 1.1 criteria.

2.2 Secondary Objectives

4. PFS (Progression Free Survival)： PFS was defined as the time from enrollment to the first documented disease progression or death from any cause.

5. Safety: AEs were evaluated, graded and recorded according to National Cancer Institute Common Terminology Criteria for Adverse Events (CTCAE), version 4.0.

6. Overall survival (OS): OS was defined as the time from enrollment to the date of death from any cause or last follow-up.

3. STUDY PLAN

This is a prospective、single-site、open-label phase II clinical study. 84 enrolled patients will receive abraxane and gemcitabine until disease progression or intolerable toxicity.

Patients will receive treatment assessment every 2 cycles of treatment until disease progression according to RECIST1.1.

4. SELECTION OF SUBJECT POPULATION

4.2 Inclusion Criteria

1. Females with age ≥18 years old；
2. Histologically identified locally advanced (unresectable) or metastatic breast cancer；
3. ECOG ≤1；
4. Adequate functions with liver, renal and bone marrow:
5. Hemoglobin (Hb) ≥90g/L, white blood cell (WBC) ≥3.5×109/L, absolute neutrophil count (ANC) ≥1.5×109/L, platelet (PLT)<100×109/L;
6. Serum bilirubin＜upper limit of normal (UNL);
7. Without liver metastasis, aspartate aminotransferase/serum glutamic oxalacetic transaminase (AST/SGOT) ， serum alanine transaminase/serum glutamic pyruvic transaminase (ALT/SGPT) and alkaline phosphatase (ALP) ≤2.5×UNL;
8. With liver metastasis, AST/SGOT, ALT/SGPT and ALP >5×UNL;
Serum creatinine ≤ 1.5×UNL;
9. Life expectancy greater than 12 weeks；
10. No severe medical history of heart, lung, liver, renal and so on;
11. ICF (Informed Consent Form) before enrollment；
12. Good compliance.

4.3 Exclusion Criteria

1. Pregnancy or breast feeding (In women at childbearing age, HCG must be examined in 14 days prior to the first administration to exclude pregnancy. If positive, ultrasound examination is necessary to exclude pregnancy);

2. Women at childbearing age who refuse contraception measures in study;

3. Treatment with radiotherapy at the axial skeleton within 4 weeks before the first treatment or has not recovered from all toxicities of previous radiotherapy;

4. Use of other clinical trial medication or participating in other clinical trial currently or within the last 4 weeks.

5. Uncontrolled brain metastases. Patients with brain metastases must be locally treated and the disease must be stable for at least one month at the time of enrolling;

Participation in any investigational drug study within 4 weeks preceding treatment start;

7. Concurrent other malignancy at other sites or previous other cancer within the last 6 years, with the exception of adequately treated in situ carcinoma of cervix uteri or basal or squamous cell carcinoma of the skin or a contralateral breast cancer;

9. Patients having a history of clinically significant cardiovascular, hepatic, respiratory or renal diseases, clinically significant hematological and endocrinal abnormalities, clinically significant neurological or psychiatric conditions

10. Serious uncontrolled infections.

6. TREATMET

ABX (Abraxane) 125 mg/m2， intravenous infusion， 30min， day 1, 8, 15, q4w,

GEM (Gemcitabine) 800 mg/m2， intravenous infusion， 30min， day 1, 8, 15, q4w, until disease progression or intolerable toxicity.

6. CONCOMITANT MEDICATIONS

6.1 Premedication

5-Hydroxytryptamine-3 Receptors Antagonist, dexamethasone or promethazine can be used to prevent and relieve vomiting after chemotherapy according to doctors' decision.

6.2 Supporting treatment

All patients can receive standard supporting treatment, including blood transfusion or anti-biotics.

6.2.1 Colony-stimulating factor

Granulocyte colony-stimulating factor (G-CSF) can be used for neutropenia, but not as a preventive medication.

6.2.2 Platelet transfusion

When grade II or high platelet count decrease appears, platelet transfusion, IL-11 or thrombopoietin.

6.2.3. Bisphosphonates

Bisphosphonates for bone metastases confirmed by radiological evidence or ECT at baseline are allowed. Use of bisphosphonates after clinical trial entry with no bone metastases evidence at baseline is considered as disease progression.

6.2.4. Other concomitant medications

Other concomitant medications should be minimized. But when it comes to the interests of the patients and does not interfere with the study, it can be determined by the principal investigators. All concomitant medications used within 4 weeks before first administration should be recorded in the case report form.

Following medication is not recommended to use, except that it is necessary to use in patients:

- Medications with potential inducing effects of CYP2C8 and CYP3A4: rifampicin, carbamazepine, diphenylhydantoin sodium, efavirenz；
- Medications with potential depressing effects of CYP2C8 and CYP3A4: erythromycin, fluoxetine, gemfibrozil;

8. DOSE MODIFICATION AND TREATMENT DELAY

7.4 Treatment of adverse events and principal of dose modification

If ANC≤1.5 x 10^9/L and PLT<100 x10^9/L on day1 of every cycle of chemotherapy, the chemotherapy should be delayed. Treatment should be interrupted for

non-hematological toxicities of any grade II above (except alopecia, nausea and vomiting) until it recovers to grade II or less. The duration of chemotherapy delay is permitted up to 14 days to recover from hematological and non-hematological toxicities. Dose reduction is permitted up to twice in all courses. Abraxane is reduced

to 100mg/m^2 first, and to 80mg/m^2 secondly. Gemcitabine is reduced to 600mg/m^2 first, and to 450mg/m^2 secondly. On day 8 or day 15 of every cycle, if patient's blood test doesn't reach ANC≤1.5 x 10^9/L or PLT<80 x10^9/L, the injection of day 8 or day 15 should be delayed to 1 week at most. If the blood test doesn't still reach criteria, quit

this injection and refer to the following principals to modify the dose of chemotherapy.

7.5 Reduction of both two drugs

1. Grade III/IV febrile neutropenia occur(with a single temperature of >38.3℃ or a sustained temperature of ≥38 ℃ for more than one hour)；
2. Grade IV neutropenia for more than 7 days;
3. Other situations that investigators decide to reduce the dose;

7.6 Reduction of Abraxane

5. If grade II or above neurologic toxicity occurs, patients should take the

electromyogram to confirm and receive symptomatic treatments. Treatment should be interrupted for 14 days at most until it recovers to grade II or less, and dose should be reduced according to principal of dose modification. If it doesn't recover to grade II or less, single gemcitabine is permitted in the subsequent treatments；

6. Except alopecia, nausea and vomiting, other severe non-hematological toxicities, which are considered related to Abraxane, doctors can reduce the dose of Abraxane according to principal of dose modification.

7.5 Reduction of Gemcitabine

1. Grade III/IV platelet count decreased or decreased platelet related hemorrhage
2. If grade II or above lung toxicities occur, and are supposed to be related to gemcitabine, gemcitabine should be permanently forbidden in this patient.
3. Hemolytic uremic syndrome，HUS: A disorder characterized by a form of thrombotic microangiopathy with renal failure, hemolytic anemia, severe thrombocytopenia, and increased bilirubin or LDH. HUS should be considered first, and gemcitabine should be stopped.
4. Except alopecia, nausea and vomiting, other severe non-hematological toxicities, which are considered related to gemcitabine, doctors can reduce the dose of Abraxane according to principal of dose modification.

8. TREATMENT DISCONTINUATION

Patients will discontinue the study treatment and follow-up until death under the following circumstances:

8. Disease progression during the study;
9. Patients who are still intolerable after twice dose modification;
10. Treatment are interrupted for more than 2 weeks;
11. Pregnancy;

5. Protocol violations that render the patient unsuitable for further treatment ;
6. Any other reason deemed appropriate by the investigator.

9. ESTIMATION OF SAMPLE SIZE

The ORR of combination was hypothesized as 55 % with the addition of gemcitabine in the whole population compared with 40 % for weekly nab-paclitaxel monotherapy, as reported in previous studies with 80 % power, type 1 error, α=0.05. 84 patients were required in consideration of 10 % expulsion rate.

10. MEASUREMENTS OF STUDY VARIABLES

10.1 Tumor assessment

Radiographic assessment (CT or MRI) and tumor markers for disease evaluation should be performed within 4 weeks prior to the treatment, with QOL questionnaire FACT-G+B+TAXANE (version 4). RECIST 1.1 is used to evaluate efficacy, including measurable and unmeasurable lesions, target or non-target lesions. Objective tumor assessment is evaluated as complete response (CR), partial response (PR), stable disease (SD) and disease progression (PD).

Baseline assessment should be performed within 4 weeks prior to the treatment, and as near as possible. Radiographic assessment includes chest, abdomen, pelvis CT or MRI. Measurement of tumor assessment in follow-up visits should be the same with the baseline. Other suspected lesions may be evaluated at baseline and follow-up visits. After baseline assessment, all enrolled patients should receive the response assessment and QOL questionnaire every two cycle ± 3 days, until disease progression occurs according to RECIST 1.1. If patients receive a response assessment not in pre-set time, next response assessment should be administered as the pre-set time, as long as the patients still enroll in this study. Efficacy of every patients who reach ORR (CR+PR) should be confirmed after at least 4 weeks. Interval of next response assessment should be 2 courses of treatment.

If the patient withdraws the study before disease progression (and/or receive other

treatment besides the study), the patient should be followed up until disease progression according to RECIST 1.1.

Objective tumor assessment is evaluated as complete response (CR), partial response (PR), stable disease (SD) and disease progression (PD) according to RECIST 1.1. Evaluation of target lesion as PD should be at least a 20% increase in the sum of diameters, taking as reference the smallest sum on study. Except PD, other response (CR, PR and SD) should be compared with the baseline.

If the investigator and clinical research coordinator cannot confirm progression, especially response of non-target lesion and emerging of new lesion, the treatment can continue until next evaluation, or a quick re-evaluation dependent on clinical practice. If disease progression is confirmed in re-evaluation, the date of disease progression should be the time that response is first suspected as PD.

If disease progression is dependent on the progression of non-target disease, there must be an overall level of substantial worsening in non-target disease such that, even in presence of SD or PR in target disease, the overall tumour burden has increased sufficiently to merit discontinuation of therapy. A modest 'increase' in the size of one or more non-target lesions is usually not sufficient to quality for unequivocal progression status.

After disease progression, follow-up visits should be every 3 month until death of patients.

10.2 Evaluation of adverse event

Adverse events will be collected after every cycle of chemotherapy, according to the physical examination and laboratory assessment. Adverse events were graded using the National Cancer Institute Common Terminology Criteria for Adverse Events version 4.0.

11. ADVERSE EVENT

11.1 Definitions

An adverse event is the development of an undesirable medical condition or the deterioration of a pre-existing medical condition following or during exposure to a

pharmaceutical product, whether or not considered causally related to the treatment.

11.2 Evaluation of Adverse Event

Adverse Events will be collected throughout the treatment period. Every adverse event should be documented in the CRF, whether or not considered causally related to the treatment.

11.3 Definition of Serious Adverse Event (SAE)

A serious adverse event is an AE occurring during any study phase and fulfils one or more of the following criteria:

- results in death
- is immediately life-threatening
- requires in-patient hospitalisation or prolongation of existing hospitalisation
- results in persistent or significant disability or incapacity
- is a congenital abnormality or birth defect

Adverse Event is an important medical event that may jeopardise the subject or may require medical intervention to prevent one of the outcomes listed above. If any SAE occurs in the course of the study, then Investigators or other site personnel should inform immediately.

11.4 Grading of Adverse Event

Adverse event is graded as Grade 1-4 by National Cancer Institute Common Terminology Criteria for Adverse Events version 4.0. If there is no grading according CTCAE 4.0, adverse event can be evaluated as follows:

Mild: mild symptoms; intervention not indicated; no influence on ADL;

Moderate: moderate symptoms; minimal, local or noninvasive intervention indicated; no influence on ADL;

Severe: medically significant but not immediately life-threatening; limiting ADL;

Life-threatening consequences: life-threatening and need urgent intervention; or death

related to AE.

11.5 Reporting of Adverse Event

The following variables will be collected for each AE: AE (verbatim), the date when the AE started and stopped, intensity, whether the AE is serious or not, action taken with regard and outcome. Investigators or other site personnel report as requirements of SFDA. In this study, SAE should be reported to principal investigator, GCP, ethics immediately but no later than 24 hours.

References:

1. Jemal, A. *et al.* Cancer statistics, 2007. *CA Cancer J Clin* **57**, 43-66 (2007).
2. van Zuylen, L., Verweij, J. & Sparreboom, A. Role of formulation vehicles in taxane pharmacology. *Invest New Drugs* **19**, 125-141 (2001).
3. Ibrahim, N.K. *et al.* Phase I and pharmacokinetic study of ABI-007, a Cremophor-free, protein-stabilized, nanoparticle formulation of paclitaxel. *CLIN CANCER RES* **8**, 1038-1044 (2002).
4. Desai, N. *et al.* Increased antitumor activity, intratumor paclitaxel concentrations, and endothelial cell transport of cremophor-free, albumin-bound paclitaxel, ABI-007, compared with cremophor-based paclitaxel. *CLIN CANCER RES* **12**, 1317-1324 (2006).
5. Henderson, I.C. & Bhatia, V. Nab-paclitaxel for breast cancer: a new formulation with an improved safety profile and greater efficacy. *Expert Rev Anticancer Ther* **7**, 919-943 (2007).
6. Roy, V., LaPlant, B.R., Gross, G.G., Bane, C.L. & Palmieri, F.M. Phase II trial of weekly nab (nanoparticle albumin-bound)-paclitaxel (nab-paclitaxel) (Abraxane) in combination with gemcitabine in patients with metastatic breast cancer (N0531). *ANN ONCOL* **20**, 449-453 (2009).
7. Lobo, C. *et al.* Final results of a phase II study of nab-paclitaxel, bevacizumab, and gemcitabine as first-line therapy for patients with HER2-negative metastatic breast cancer. *Breast Cancer Res Treat* **123**, 427-435 (2010).
